# mSphere of Influence: Going Native, or the Risk of Overreliance on Recombinant Antigens

**DOI:** 10.1128/mSphere.00224-20

**Published:** 2020-04-08

**Authors:** Mary Lopez-Perez

**Affiliations:** a Centre for Medical Parasitology, Department of Immunology and Microbiology, University of Copenhagen, Copenhagen, Denmark

**Keywords:** antibodies, Colombia, malaria, PfEMP1, pregnancy, native proteins, recombinant proteins, VAR2CSA

## Abstract

Mary Lopez-Perez works on immunology and pathogenesis of malaria. In this mSphere of Influence article, she reflects on how the paper “Functional antibodies against VAR2CSA in nonpregnant populations from Colombia exposed to Plasmodium falciparum and Plasmodium vivax*”* by S. Gnidehou, J. Doritchamou, E. M. Arango, A. Cabrera, et al. (Infect Immun 82:2565−2573, 2014, https://doi.org/10.1128/IAI.01594-14) made her cautious of relying exclusively on recombinant proteins when evaluating antibody responses.

## COMMENTARY

Plasmodium falciparum, followed by Plasmodium vivax, is the parasite species responsible for most of the malaria cases worldwide every year, with 228 million cases estimated in 2018 (1). Children under 5 years old and pregnant women are the most vulnerable groups affected by malaria, since they lack specific protective immunity against the parasites. This protection is mainly antibody mediated and predominantly targets P. falciparum erythrocyte membrane protein 1 (PfEMP1), the major antigen expressed on the infected erythrocyte (IE) surface (2). The susceptibility of pregnant women to malaria is related to VAR2CSA (3), a particular PfEMP1 variant that binds to an unusually low-sulfated form of chondroitin sulfate A (CSA) found in the placental intervillous spaces, allowing the selective accumulation of IEs in the placenta (4, 5). Therefore, it is generally assumed that parasites expressing this variant do not thrive in nonpregnant individuals. Indeed, the levels of VAR2CSA-specific IgG in plasma increase with parity and inhibit IEs binding to CSA. These antibodies are restricted to pregnancy, with only a few reports of low levels among P. falciparum-exposed men, children, and nulligravidae. This fact and the identification of the CSA-binding domain in VAR2CSA constitute the rationale for the development of a VAR2CSA-based vaccine to protect against placental malaria. Such vaccines are currently being tested in phase 1a/1b clinical trials (ClinicalTrials.gov. NCT02647489 and NCT02658253).

Since the intensity of P. falciparum transmission in Colombia is low, malaria during pregnancy, including placental malaria, occurs infrequently (6–8). It was therefore highly unexpected when Gnidehou et al. (9) reported high levels and frequencies (50 to 70%) of VAR2CSA-specific IgG in Colombia, not only in pregnant women with or without malaria infection but also among nulligravidae, men, and children living in areas of the country where malaria is endemic. To evaluate the antibody levels, Gnidehou et al. (9) had used recombinant domains (DBL3X, DBL5ε, and ID1-ID2) of VAR2CSA produced in baculovirus-infected Sf9 insect cells (referred to as BIC). Their data showed that men and children from Colombia had higher antibody levels to the ID1-ID2 domain than pregnant women. In contrast, their control samples from Benin (men, children, and nulligravidae women) had antibodies below the cutoff level against all the domains, in agreement with numerous reports from Africa. The authors proposed that the high VAR2CSA reactivity among Colombians was related to cross-reactive antibodies induced by the P. vivax antigen PvDBP (10). Both P. falciparum and P. vivax are transmitted in Colombia, whereas P. vivax transmission is very low in Africa (1). Thus, the findings pointed to a novel and unanticipated mode of acquisition of VAR2CSA-specific IgG in Colombia. Indeed, the report not only suggested that the current understanding of immunity to placental malaria needed to be revised for areas where several malaria parasite species are cotransmitted but called the rationale underpinning the development of VAR2CSA-based vaccines against placental malaria into question.

To shed additional light on the prevalence and specificity of IgG recognizing VAR2CSA in this population (11), we decided to measure antibody response in a different set of samples from Colombia, using a full-length recombinant VAR2CSA (FV2_BIC_) expressed in the same system used by Gnidehou et al. (9). Our samples included Colombian pregnant women, children, and men with malaria or previously exposed to the disease and a control group of nonpregnant malaria-exposed women from Ghana. In agreement with Gnidehou and colleagues, we found that FV2_BIC_-specific IgG antibodies were frequent among pregnant and nonpregnant individuals. Surprisingly, we found that the FV2_BIC_-specific IgG levels in the Colombian samples were positively correlated with IgG levels to another PfEMP1 full-length recombinant protein (FV6_BIC_), expressed in the same system but a variant not restricted to parasites infecting pregnant women. It was surprising because such a correlation would not be expected intuitively and because it was not observed in samples from Ghana or in individuals exposed exclusively to P. vivax, arguing against the idea of species cross-reactive antibodies as the explanation. Moreover, levels of IgG recognizing the recombinant VAR2CSA protein did not show the expected correlation with IgG to the corresponding native protein expressed on the surfaces of IEs. It thus appeared that the Colombian plasma samples contained IgG recognizing epitopes in the recombinant proteins that are absent from the native counterpart—epitopes that might not even be specific to malaria. To test this, we expressed the FV2 protein in mammalian CHO cells (FV2_CHO_) rather than in insect cells. Indeed, the levels of IgG to FV2_CHO_ were very low in plasma from nonpregnant Colombian individuals and did not correlate with the levels of FV2_BIC_-specific IgG. Furthermore, they positively correlated with the native protein only among pregnant women. Finally, the IgG reactivity to FV2_BIC_ could not be depleted by incubation with FV2_CHO_.

We did not pursue the identity of the epitopes in the FV2_BIC_ proteins recognized by samples from Colombia, as we considered that beyond the scope of our work. However, our investigation did not yield support for the suggestion by Gnidehou et al. (10) that PvDBP, a P. vivax antigen, induces cross-reactive antibodies that recognize VAR2CSA. Thus, our data show that responses to recombinant proteins can be seriously misleading if not checked carefully against data obtained with the corresponding native proteins. An independent report (12) of remarkable differences in the ability of native and recombinant PfEMP1 proteins to bind to endothelial receptors implicated in malaria pathogenesis emphasizes this caveat.
